# Uveitic glaucoma-like features in *Yap* conditional knockout mice

**DOI:** 10.1038/s41420-023-01791-6

**Published:** 2024-01-25

**Authors:** Juliette Bitard, Elodie-Kim Grellier, Sophie Lourdel, Helena Prior Filipe, Annaïg Hamon, François Fenaille, Florence Anne Castelli, Emeline Chu-Van, Jérôme E. Roger, Morgane Locker, Muriel Perron

**Affiliations:** 1 Université Paris-Saclay, CNRS, Institut des Neurosciences Paris-Saclay, Saclay, France; 2 West Lisbon Hospitals Center, Hospital de Egas Moniz, Lisbon, Portugal; 3 Egas Moniz Center for Interdisciplinary Research, Lisbon, Portugal; 4 Université Paris-Saclay, CEA, INRAE, Département Médicaments et Technologies pour la Santé (DMTS), MetaboHUB, Gif sur Yvette, France

**Keywords:** Cell death in the nervous system, Glial biology, Neurodegeneration

## Abstract

Glaucoma is a multifactorial neurodegenerative disease characterized by the progressive and irreversible degeneration of the optic nerve and retinal ganglion cells. Despite medical advances aiming at slowing degeneration, around 40% of treated glaucomatous patients will undergo vision loss. It is thus of utmost importance to have a better understanding of the disease and to investigate more deeply its early causes. The transcriptional coactivator YAP, an important regulator of eye homeostasis, has recently drawn attention in the glaucoma research field. Here we show that *Yap* conditional knockout mice (*Yap* cKO), in which the deletion of *Yap* is induced in both Müller glia (i.e. the only retinal YAP-expressing cells) and the non-pigmented epithelial cells of the ciliary body, exhibit a breakdown of the aqueous-blood barrier, accompanied by a progressive collapse of the ciliary body. A similar phenotype is observed in human samples that we obtained from patients presenting with uveitis. In addition, aged *Yap* cKO mice harbor glaucoma-like features, including deregulation of key homeostatic Müller-derived proteins, retinal vascular defects, optic nerve degeneration and retinal ganglion cell death. Finally, transcriptomic analysis of *Yap* cKO retinas pointed to early-deregulated genes involved in extracellular matrix organization potentially underlying the onset and/or progression of the observed phenotype. Together, our findings reveal the essential role of YAP in preserving the integrity of the ciliary body and retinal ganglion cells, thereby preventing the onset of uveitic glaucoma-like features.

## Introduction

Glaucoma is a multifactorial neuropathy characterized by optic nerve damage and progressive loss of retinal ganglion cells (RGCs). The etiological diversity of the disease, which leads to more than ten clinical subtypes (e.g. open-angle glaucoma, normal tension glaucoma, uveitic glaucoma, pigmentary glaucoma, etc.), as well as the very limited number of animal models, have hindered our understanding of the underlying pathological mechanisms. Dysfunctions of both the anterior chamber and the neuroretina are important components of glaucoma etiology. In the anterior chamber, inflammation of the uvea (i.e. iris, choroid, ciliary body) called uveitis, pigment dispersion from the iris, and increased intraocular pressure (IOP) constitute well-known risk factors. High IOP results from the imbalance between the production of the aqueous humor by the non-pigmented epithelium (NPE) of the ciliary body, and its drainage by the trabecular meshwork in the iridocorneal angle [1]. Since IOP is the only modifiable factor in glaucoma, it constitutes the target of first-line medications. Nevertheless, between 30 and 90% of glaucomatous patients will not present this trait [2], suggesting the existence of other pathogenic processes. In line with this, both normal and high-tension glaucoma are accompanied at the neuroretinal level by chronic gliosis, oxidative stress, and vascular dysfunctions [3]. These pathological features might be explained, at least in part, by Müller glial cell dysfunction [4–6]. Among the fundamental roles of Müller cells is the regulation of extracellular glutamate levels through its recycling into glutamine that further fuels neurons [7]. In several experimental models of glaucoma, an increase in extracellular glutamate levels was reported to contribute to RGC death [7].

Despite major breakthroughs in the global understanding of glaucoma, the very limited number of animal models impedes further dissection of the complex etiology of this group of diseases. The Hippo pathway effector YAP recently emerged as an orchestrator of eye homeostasis [8]. We and others showed that its haploinsufficiency leads to cataract and age-related photoreceptor degeneration [9, 10]. With regards to the glaucoma research field, a genome-wide association study identified *Yap* as a new risk locus for primary open-angle glaucoma [11]. In line with this, YAP is expressed in several ocular cell types, including those affected in glaucoma (i.e. the NPE of the ciliary body, the trabecular meshwork, and Müller cells) [12]. Functional studies revealed its contribution to human trabecular meshwork cell stiffening in vitro, through its mechanotransducer function [13, 14]. This further substantiates the idea that YAP dysfunction might play a role in glaucoma, since changes in mechanical trabecular meshwork properties are proposed to underlie the increased resistance to aqueous humor outflow, and thereby to participate in the IOP elevation observed in glaucomatous patients [15]. Whether altered *Yap* expression in NPE cells of the ciliary body and/or in Müller glial cells could contribute to glaucoma development has not been investigated yet. Here, taking advantage of *Yap* conditional knockout mice that we previously described [16], we demonstrate that selective loss of YAP in both Müller cells and the ciliary body triggers uveitic glaucoma-like pathological features. Overall, our study suggests that deregulation of this cofactor may favor glaucoma onset and/or progression in humans.

## Results

### The ciliary body progressively collapses with age in *Yap* cKO mice

**Fig. 1:**
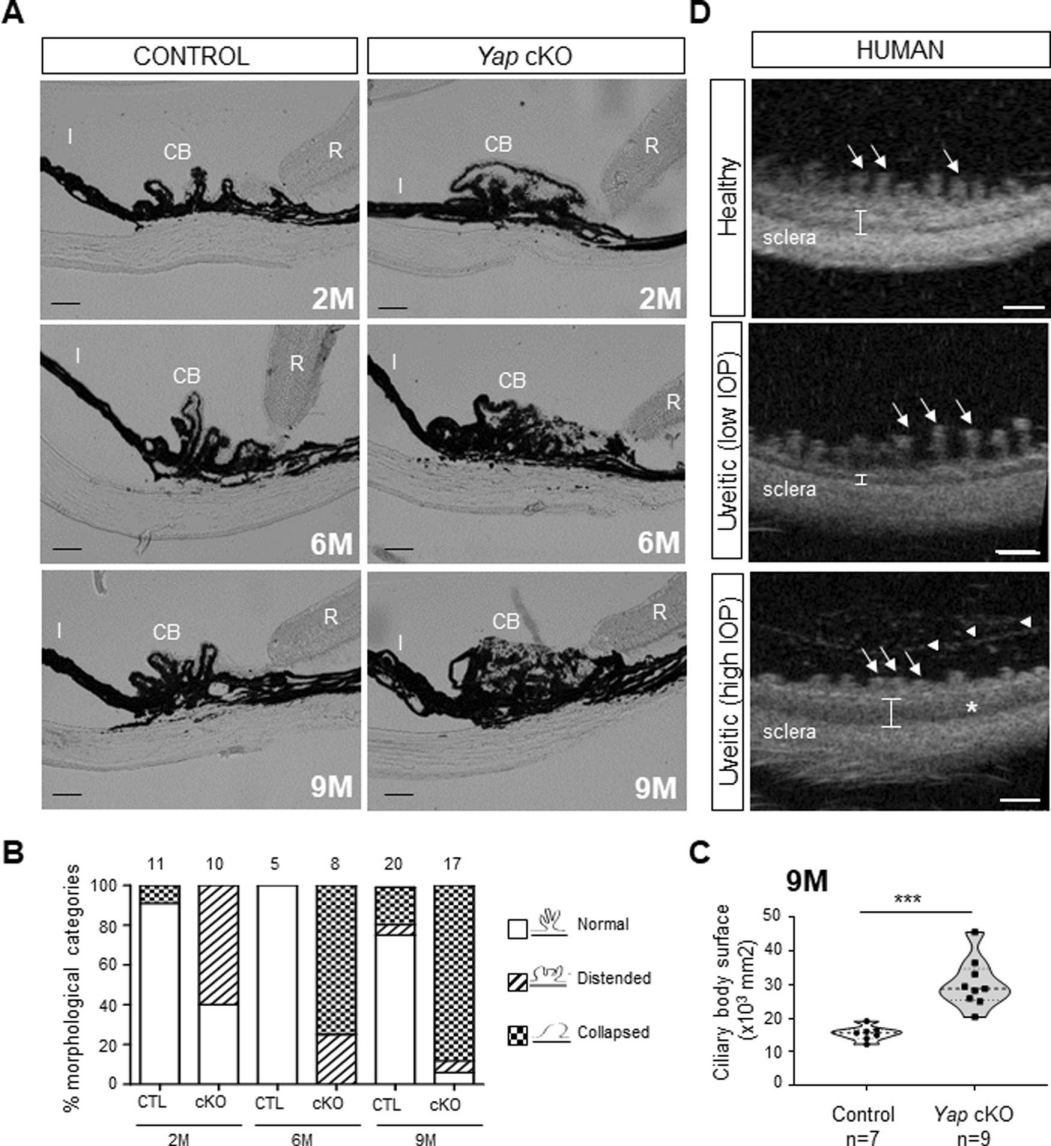
The ciliary body of *Yap* cKO mice progressively collapses as observed in human patients with uveitis. **A** Brightfield micrographs from 2-, 6-, and 9-month-old (2 M, 6 M, 9 M) control (*Yap*^*flox/flox*^) and *Yap* cKO ciliary body sections. CB: Ciliary body, I: Iris, R: Retina. Scale bars: 50 mm. **B** Quantification of control (CTL) and *Yap* cKO eyes harboring normal or defective ciliary bodies. We defined the ciliary body morphology according to the shape of its processes, which were either “distended” with a loss of clearly defined separation between the non-pigmented and the pigmented epithelium, or “collapsed” when the processes had completely disappeared. The number of eyes analyzed for each condition is indicated above the stacked histograms. **C** Quantification of the ciliary body surface in 9-month-old control and *Yap* cKO individuals. In these violin-plots, the thick dotted line represents the median, and the thin dotted lines the interquartiles. The number of analyzed ciliary bodies in each condition is indicated below the x-axis. Statistics: Mann-Whitney test, ***p ≤ 0.001. **D** Transverse section images from human healthy individuals and patients with chronic uveitis taken with an ultrasound biomicroscope. Compared to the healthy individual, the patient with uveitis presenting with low IOP shows atrophic (almost absent) pars plicata, thinner ciliary muscle area (white bracket), and elongated ciliary processes (arrows). The second patient with uveitis and high IOP presents with pars plicata edema (asterisk), increased thickness of ciliary muscle area (bracket), and shortened ciliary processes (arrows). White arrowheads point to putative inflammatory cells and fibrin deposition Scale bars: 1 mm.

In order to evaluate the impact of *Yap* deletion in both Müller cells and the ciliary body, we used *Yap*^*flox/flox*^*;Rax-Cre*^*ERT2*^ mice (*Yap* cKO) that we previously generated [16]. In the postnatal or adult eye, the Cre recombinase expression pattern is restricted to Müller glia and NPE cells of the ciliary body, where YAP was previously shown to be expressed [16, 17]. *Yap* deletion was induced at postnatal day 10 (P10), when both Müller and NPE cells are already differentiated [18, 19]. We first assessed ciliary body morphology at different ages. Two months after *Yap* deletion induction, around 60% of the animals exhibited distended ciliary processes, indicative of cell-cell contact loss (Fig. 1A). The ciliary processes thereafter further regressed, with complete collapse in 100% of *Yap* cKO mice progressively occurring between six and nine months of age, without angle closure (Fig. 1A, B). Confirming their dramatic disorganization, ciliary bodies of 9-month-old *Yap* cKO mice exhibited an increased surface compared to age-matched controls (Fig. 1C). In humans, such alterations of the ciliary body have only been described in patients with uveitis [20, 21]. We thus next sought to further characterize the ciliary body defects of such patients. Interestingly, clinical examination using ultrasound biomicroscopy revealed distension (low IOP patients) or collapse (high IOP patients) of ciliary body processes (Fig. 1D), as observed in *Yap* cKO mice. Overall, these data demonstrate that YAP is required for the structural maintenance of the ciliary body. They also interestingly highlight a ciliary body phenotypic resemblance between *Yap* cKO mice and patients with uveitis.

### *Yap* cKO mice show alterations of the iridal blood-aqueous-barrier

**Fig. 2:**
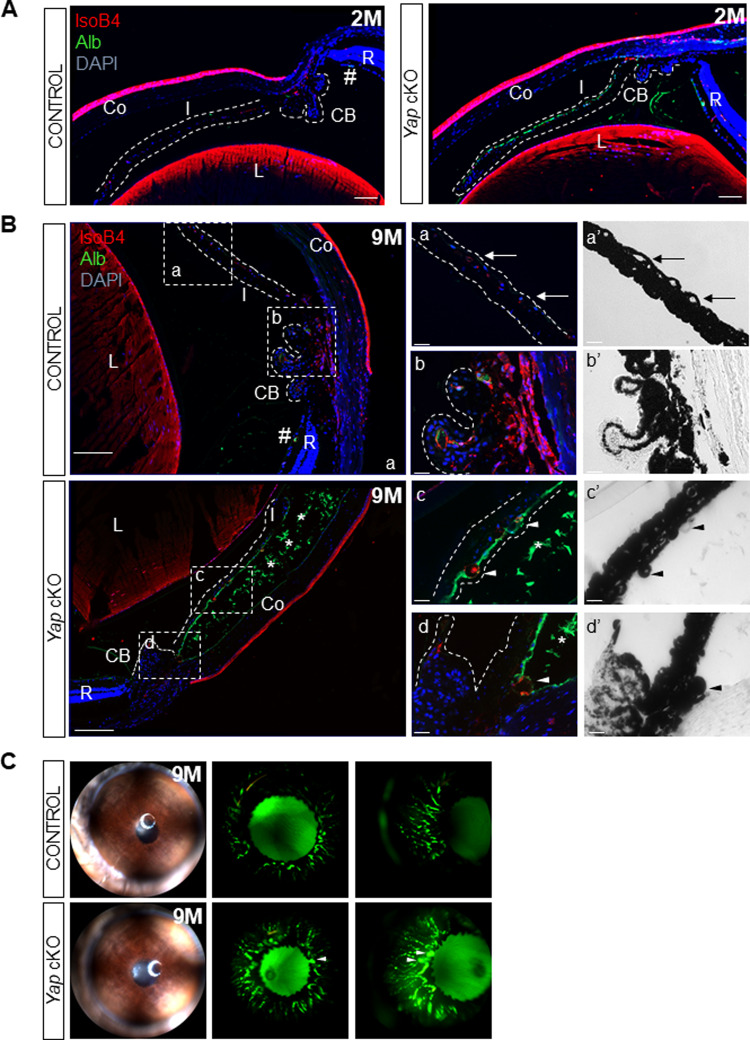
*Yap* cKO eyes exhibit a breakdown of the blood-aqueous barrier. **A**, **B** Sections from control (*Yap*^*flox/flox*^) and *Yap* cKO eyes at 2 (2 M, *n* = 4; A) or 9 months of age (9 M, *n* = 4; B). Sections were stained with IsoB4 (red) to assess blood vessel wall integrity and with anti-albumin antibody (green) to assess blood leakage from capillaries. Nuclei were counterstained with DAPI (blue). Dashed white lines delineate the iris and the ciliary body. Hashtag points to an intact blood vessel harboring albumin staining exclusively inside the lumen. Regions inside the dashed white squares are depicted at a higher magnification on the right panels. Shown are either fluorescence (a, b, c, d) or brightfield (a’, b’, c’, d’) images. In panels a, a’, arrows point to normal iridal blood vessels. In c, c’, d, d’, asterisks indicate proteinaceous exudates, while arrowheads indicate abnormal blood vessels. CB: Ciliary body, Co: Cornea, I: Iris, L: Lens, R: Retina. Scale bars: left panels, 100 mm; right panels, 20 mm. **C** Fundus imaging and fluorescein angiography (en-face and profile views) of 9-month-old control (*n* = 5) and *Yap* cKO (*n* = 7) iris. Arrowheads indicate microaneurisms.

We next sought other signs of uveitis in *Yap* cKO eyes, such as a breakdown of the blood-aqueous barrier (BaB), which results in exudation of blood components like albumin and which is usually accompanied by fibrosis. We first examined albumin immunostaining as a readout of blood barrier defects [22]. In control animals, albumin staining was restricted to blood vessels and was only detectable in the interior space of the biggest ones (Fig. 2A, B). In contrast, we observed albumin staining outside vessel walls in all *Yap* cKO eyes, suggesting iridal blood leakage as early as two months of age (Fig. 2A). In aged *Yap* cKO eyes, Isolectin-B4 (IsoB4)-labeled iridal blood vessel walls exhibited signs of degeneration compared to control ones, appearing either dilated (rounded blood vessel, Fig. 2B-c,c’) or degraded (unpigmented blood vessel, Fig. 2B-c,c’). In addition, albumin-labeled proteinaceous exudates (asterisks in Fig. 2B) likely emanating from the iridal endothelial cell layer were observed, confirming tissue damage. To further substantiate BaB disruption at the iris level, we performed fluorescein angiography on 9-month-old mice (Fig. 2C). Compared with control eyes, where iridal vascularization consists of thin regular blood vessels, *Yap* cKO ones exhibited thicker blood vessels, highly visible at the arcade of the pupillary margin and covering a larger vascular area. They also displayed blood leakage as indicated by iridal zones presenting diffuse fluorescein staining. Taken together, our results demonstrate that loss of blood vessel integrity in the iris constitutes an early feature of *Yap* cKO phenotype.

### *Yap* cKO mice harbor glaucoma-like features

**Fig. 3:**
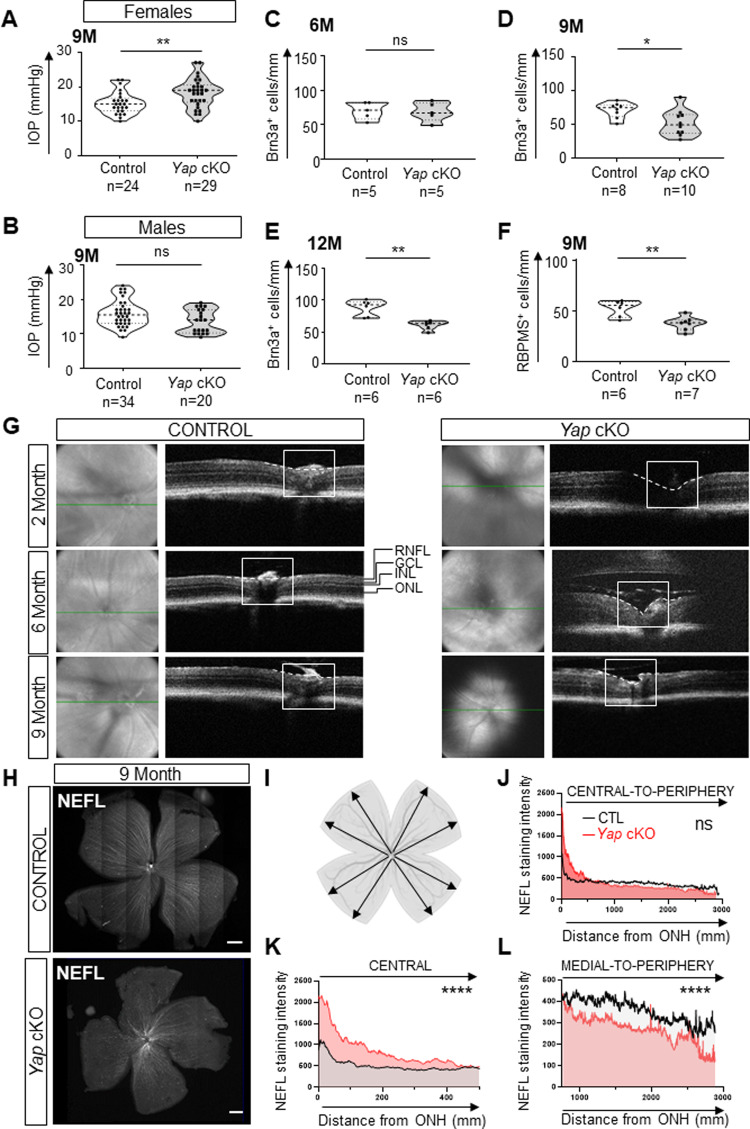
*Yap* cKO mice harbor glaucoma-like features. **A**, **B** Measurement of intraocular pressure (IOP) in female and male eyes from 9-month-old control (*Yap*^*flox/flox*^) and *Yap* cKO retinas. **C**–**F** Quantification of RGCs (Brn3a- **C**–**E** or RBPMS- **F** positive cells) in control and *Yap* cKO retinas at 6-, 9-, or 12 months of age as indicated (6 M, 9 M, 12 M). In violin plots, the thick dotted line represents the median, and the thin dotted lines the interquartiles. The number of analyzed eyes in each condition is indicated below the x-axis. **G** Representative OCT B-scan (right) corresponding to the green line shown on the en face image, and en face (left) images of 2-, 6-, and 9-month-old (2 M, 6 M, 9 M) control and *Yap* cKO retinas. The ONH is delineated by a white square and the surface of the retina at the ONH level is highlighted with a dashed line. Similar defects were observed in at least 3 individuals per condition. GCL ganglion cell layer, INL inner nuclear layer, ONL outer nuclear layer, RNFL retinal nerve fiber layer. **H**–**L** Immunostaining analysis of NEFL expression in 9-month-old control (*n* = 3) and *Yap* cKO retinas (*n* = 6). **H** Representative images of flat-mounted retinas. Scale bars: 500 mm. **I**–**L** Quantifications of NEFL staining intensity. Black arrows in the schematic in **I** represent the eight measurements performed in each analyzed retina (intensity measured every 0.645 mm starting from the ONH) using the Zen software. **J** Mean intensity along the central to peripheral axis. **K**, **L** are enlarged representations of intensity values measured either in the central part of the retina **K** or in the medial to peripheral region **L**. Statistics: Mann-Whitney test, ns: non-significant, *p ≤ 0.05, **p ≤ 0.01, ****p < 0.0001.

The BaB breakdown and ciliary body collapse occurring in *Yap* cKO mice are reminiscent of the phenotype reported in the widely used glaucoma mouse model DBA/2 J [23, 24]. This led us to hypothesize that *Yap* cKO individuals might also develop glaucomatous features with age. Considering the essential role of the NPE in aqueous humor production, we examined whether the collapse of *Yap* cKO ciliary body might be associated with abnormal IOP (i.e. imbalance between inflow and outflow of aqueous humor). When IOP was assessed without gender consideration, no significant difference was detected between control and *Yap* cKO eyes, neither at 6 nor at 9 months of age (Supplementary Fig. 1A, B), nor even at 12 months of age (data not shown). In the DBA/2J model, the IOP raise occurs earlier and at higher rates in females compared to males, despite an apparent absence of histological differences in CB collapse [23, 25]. Therefore, we re-examined IOP values taking the gender into account. The comparison with sex-matched controls revealed no difference for 9-month-old male *Yap* cKO eyes, but an increased IOP for female *Yap* cKO (Fig. 3A, B). Thus, although the onset and progression of *Yap* cKO CB structural abnormalities are not gender-specific (100% of cKO mice exhibit CB collapse), the consequences on the inflow/outflow balance appears to be more severe in females than in males, as described in DBA/2J eyes.

We next further examined potential glaucomatous features in *Yap* cKO eyes by evaluating the number of RGCs. We first used Brn3a as a marker, since it is expressed in ∼90% of RGCs [26]. At 6 months of age, the proportion of Brn3a-labeled cells within the ganglion cell layer of *Yap* cKO mice was unchanged compared to control retinas (Fig. 3C). However, it was significantly decreased by 27 and 30% in 9- and 12-month-old individuals (Fig. 3D, E). This result was confirmed in 9-month-old *Yap* cKO retinas using an antibody against RBPMS [27], a pan-RGC marker (Fig. 3F). Of note, the pattern of RGC loss was found homogenous throughout *Yap* cKO retinas (Supplementary Fig. 2). Together, these results suggest that RGC survival is affected in an age-dependent manner in *Yap* cKO retinas.

We then turned to optic coherence tomography (OCT) to visualize the optic nerve head (ONH). Control mice harbored a uniformly flat ONH whatever their age. In contrast, *Yap* cKO ONH was found excavated (dashed white line, Fig. 3G) from 2 months onwards. Finally, 9-month-old *Yap* cKO retinas harbored signs of nerve fiber degeneration, as assessed by the decreased intensity of neurofilament light chain (NEFL) labeling throughout the retina (Fig. 3H). Of note, further spatial examination of the staining revealed significantly decreased NEFL levels in the peripheral retina compared to control mice (Fig. 3I–L). In the central retina, however, the intensity was increased, a pattern reminiscent of the observed early neurofilament accumulation that precedes RGC death in the DBA/2J mouse model of glaucoma [28]. Collectively, these data indicate that ONH excavation is an early event that precedes RGC loss and nerve fiber degeneration in *Yap* cKO mice.

### Reactive gliosis and retinal vasculature defects precede RGC death in *Yap* cKO retinas

**Fig. 4:**
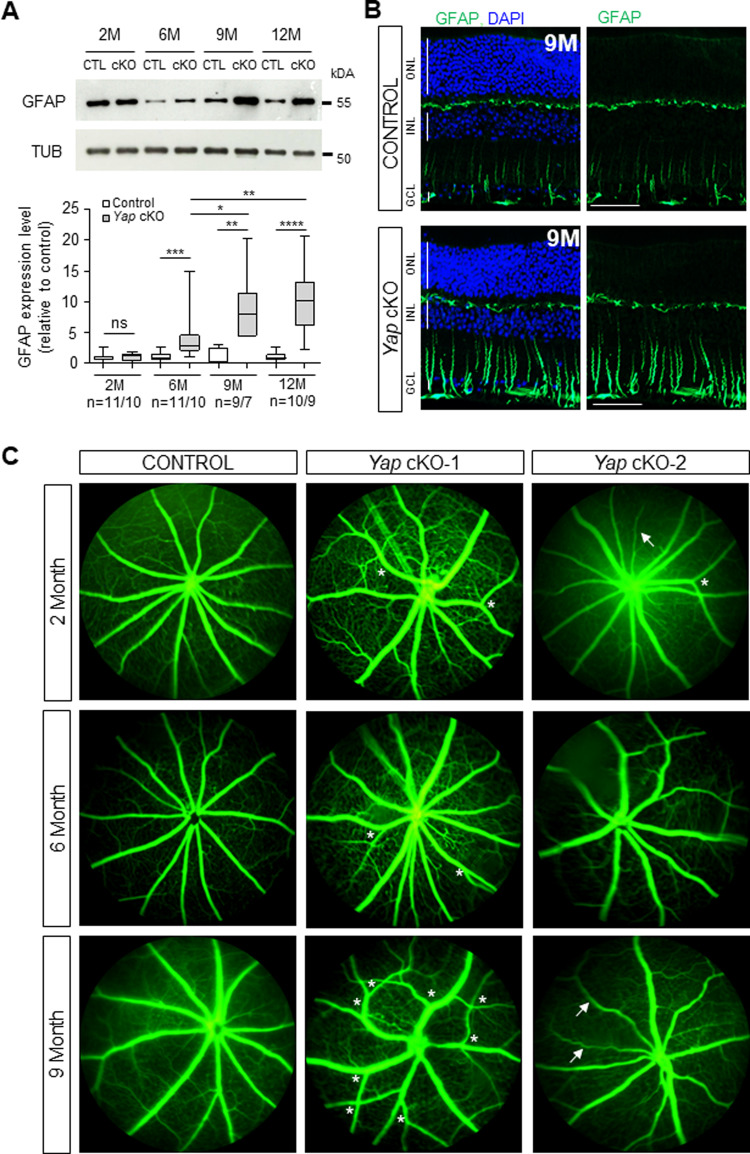
*Yap* cKO retinas exhibit reactive gliosis and vascular defects. **A** Western blot analysis of Glial Fibrillary Acidic Protein (GFAP) expression in 2-, 6-, 9-, and 12-month-old (2 M, 6 M, 9 M, 12 M) control (CTL; *Yap*^*flox/flox*^) and *Yap* cKO retinas. Expression levels were normalized to α-Tubulin (TUB) expression. The number of analyzed retinas (CTL/*Yap* cKO) is indicated below the x-axis. Statistics: Mann-Whitney test, *p ≤ 0.05, **p ≤ 0.01, *** p ≤ 0.001, **** p ≤ 0.0001, ns: non-significant. **B** Immunofluorescence analysis of GFAP expression on retinal sections from 9-month-old (9 M) control and *Yap* cKO mice. Nuclei were counterstained with DAPI (blue). GCL ganglion cell layer, INL inner nuclear layer, ONL outer nuclear layer. Scale bars: 20 mm. **C** Fluorescein angiography performed in 2-, 6- and 9-month-old control and *Yap* cKO individuals. Note the neovascularization (increased number of branches, white asterisks) and vessel degeneration (white arrows) occurring in *Yap* cKO retinas. Two *Yap* cKO individuals per age are shown.

Müller cell gliosis is a hallmark of retinal stress. It is observed in glaucoma and has been shown to participate in its physiopathology [29, 30]. Analysis of GFAP expression by both western blot and immunostaining revealed the onset of reactive gliosis in *Yap* cKO mice from 6 months of age. This phenotype further worsened in older animals (Fig. 4A, B).

Müller cell sustained gliosis is suspected to affect the blood-retinal barrier [5] and vascular alteration is correlated to the incidence of glaucoma [31]. We thus assessed the integrity of inner retinal blood vessels in *Yap* cKO retinas. Fluorescein angiography revealed retinal vasculature defects starting at 2 months of age (Fig. 4C), thus several months before the appearance of Müller cell gliosis. The phenotype was variable among mice, with defects including excessive branching (asterisk, Fig. 4C), and thin blood vessels (white arrow, Fig. 4C).

These data collectively reveal that early vascular defects appear independently of Müller cell gliosis, which occurs later on, preceding the onset of retinal ganglion cell loss.

### Glutamate recycling is impaired in aged *Yap* cKO retinas

**Fig. 5:**
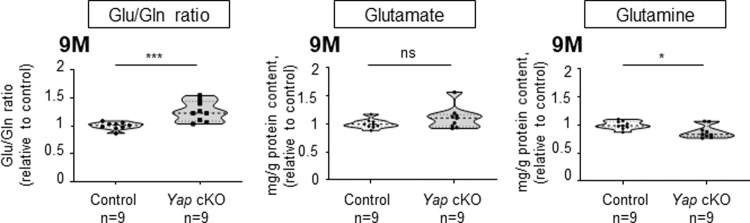
*Yap* cKO retinas show altered glutamate-to-glutamine ratios. Measurement of the glutamate-to-glutamine ratio (Glu/Gln) and glutamate or glutamine content, in 9-month-old (9 M) control and *Yap* cKO retinal extracts. The thick dotted line of violin plots represents the median, and the thin dotted lines the interquartiles. The number of analyzed retinas in each condition is indicated below the x-axis. Statistics: Mann-Whitney test, *p ≤ 0.05, ***p ≤ 0.001, ns non-significant.

**Fig. 6:**
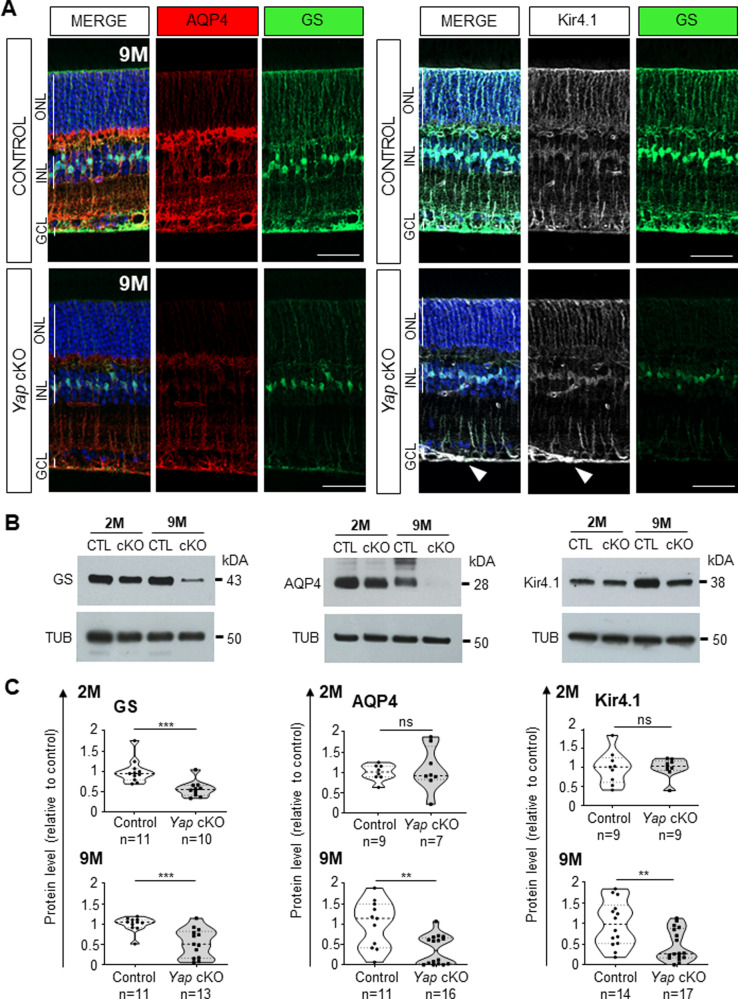
Proteins involved in glutamate recycling are affected in *Yap* cKO Müller cells. **A** Retinal sections from 9-month-old (9 M) control (*Yap*^*flox/flox*^) and *Yap* cKO retinas, immunostained for GS (in green), AQP4 (in red) and Kir4.1 (in white). Nuclei were counterstained with DAPI. The white arrowhead indicates the accumulation of Kir4.1 at the endfeet of *Yap* cKO Müller cells. GCL ganglion cell layer, INL inner nuclear layer, ONL outer nuclear layer. Scale bars: 20 mm. **B** Western blot analysis of GS, AQP4, and Kir4.1 expression in 2- and 9-month-old (2 M, 9 M) control (CTL) and *Yap* cKO retinas. **C** Corresponding quantifications. Expression levels were normalized to alpha-Tubulin (TUB) expression. The thick dotted line of violin plots represents the median, and the thin dotted lines the interquartiles. The number of analyzed retinas in each condition is indicated below the x-axis. Statistics: Mann-Whitney test, **p ≤ 0.01, ***p ≤ 0.001, ns: non-significant.

A key regulatory function of Müller cells is to ensure the recycling of glutamate, which constitutes the main excitatory neurotransmitter in the retina [32]. Control of glutamate levels is crucial for neurotransmission but also to prevent excitotoxicity-dependent RGC death, a phenomenon that has been proposed to participate in glaucoma etiology, although this is still a matter of debate [33]. We thus investigated whether RGC loss in *Yap* cKO retinas might be associated with defective glutamate recycling. We first assessed the glutamate-to-glutamine (Glu/Gln) ratio in 9-month-old retinal extracts by quantitative liquid chromatography coupled to high-resolution mass spectrometry. Aged *Yap* cKO retinas harbored a significantly higher Glu/Gln ratio compared to control ones, which results essentially from decreased levels of retinal glutamine content (Fig. 5). We then evaluated the expression level of glutamine synthetase (GS), a Müller glia-specific enzyme catalyzing the conversion of glutamate into glutamine [34]. GS levels in Müller cells were significantly decreased from two months onwards in *Yap* cKO retinas compared to control ones (Fig. 6 and Supplementary Fig. 4). This was followed in older animals by the downregulation of two other proteins involved in glutamate recycling, namely the inwardly rectifying potassium channel (Kir4.1) [32] and the water channel Aquaporin-4 (AQP4) [35, 36] (Fig. 6 and Supplementary Fig. 4). Of note, although Kir4.1 was downregulated in Müller cell processes, its expression was increased at their endfeet within the ganglion cell layer. Taken together, these results indicate an alteration of glutamate homeostasis in *Yap* cKO retinas, which most likely results from the defective expression of key Müller glia glutamate recycling proteins.

### The molecular signature of *Yap* cKO retinas

We next aimed at uncovering the early molecular mechanisms underlying the development of *Yap* cKO glaucoma-like phenotype. To this aim, we performed whole transcriptome analysis at 2 months of age and identified 276 differentially expressed genes (DEGs) between control (*Yap*^flox/flox^) and *Yap* cKO retinas (Supplementary Table S1). Importantly, 16% of these DEGs (45/276) were previously described as YAP direct target genes as retrieved in the Harmonizome database (Supplementary Fig. 3A, and [37]).

**Fig. 7:**
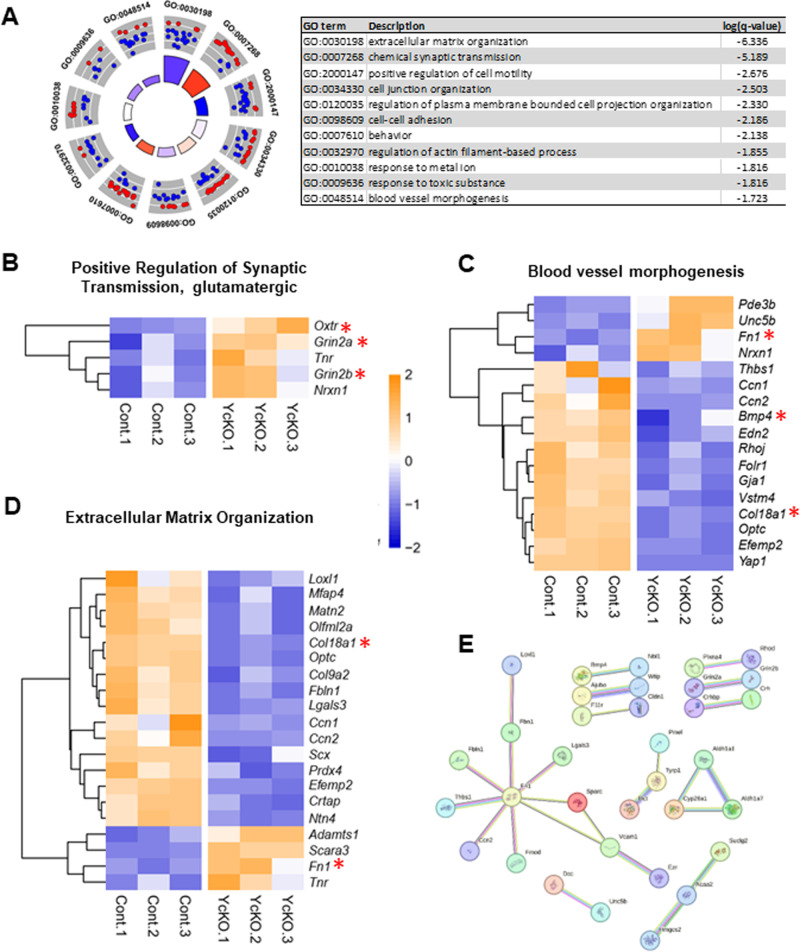
Whole transcriptome analysis of 2-month-old *Yap* cKO retinas. **A** Circular and tabular visualizations of the GO biological processes identified after pathway enrichment analysis. Blue and red dots depict down- and up-regulated genes respectively as plotted based on logFC. z score bars. **B**–**D** Hierarchical clustering analysis of the DEGs between *Yap* cKO and control retinas (filtered by fold change ≥1.5, FDR ≤ 0.05, TPM ≥ 5). Red asterisks highlight previously reported YAP direct transcriptional targets. Shown are genes related to the biological processes “positive regulation of synaptic transmission glutamatergic” (**B**), “Blood vessel morphogenesis” (**C**), and “extracellular matrix organization” (**D**) respectively. The expression pattern of each control (Cont) and *Yap* cKO retina is depicted. **E** PPI network of the 276 DEGs built with the highest confidence score ≥ 0.9.

Pathway analysis of the 276 DEGs based on Gene Ontology (GO) revealed 11 parent-enriched GO “Biological processes” (Fig. 7A–D, and Supplementary Table S2). Among the 24 DEGs belonging to the Biological Process “behavior”, 5 (*Grin2a, Grin2b, Nrxn1, Oxtr, Tnr)* are part of “positive regulation of synaptic transmission, glutamatergic” (Fig. 7B). Noteworthy, *Grin2a, Grin2b, and Oxtr* have been identified as YAP potential direct transcriptional targets (Supplementary Fig. 3A, and [37]). This not only reinforces our phenotypic observations but also points to YAP as being a potential regulator of glutamate signaling. Besides, the GO group “blood vessel morphogenesis” (17 DEGs) was also retrieved (Fig. 7C), offering a potential molecular explanation for the aforementioned blood leakage phenotype in young *Yap* cKO retinas. In this category, the *Col18a1* gene caught our attention since (i) it is a direct YAP target gene (Supplementary Fig. 3A), (ii) it is essential for retinal angiogenesis [38], and (iii) it is recognized as a genetic risk factor in one glaucoma type, namely pigmentary glaucoma [39]. This gene was also included in the GO category “Extracellular Matrix (ECM) organization” (Fig. 7D), which represented the most significantly enriched GO Biological Process in *Yap* cKO retinas. Also related to “ECM organization”, were 3 other genes (*Fbln1*, *Fn1*, and *Loxl1)* known to be associated with different types of glaucoma [39–42]. Among them, the Fibronectin-1 coding gene (*Fn1*), which expression was increased in *Yap* cKO retinas compared to control retinas, is also a direct YAP transcriptional target (Supplementary Fig. 3A). Importantly, FN1 is a major player in the development of open angle glaucoma where its accumulation in the trabecular meshwork is believed to contribute to the increase in IOP [40]. The protein-protein interaction (PPI) network of the 276 DEGs (Fig. 7E) revealed *Fn1* as the most significant hub (Node degree: 36). This result is corroborated with a recent analysis that identified this gene as one of the three major hubs among the TOP 100 DEGs in the trabecular meshwork of glaucomatous patients [43]. Finally, comparison of our dataset with that of 3-month-old DBA/2 J retinas revealed 23% (65/276) of identical DEGs. Of note, 100% (7/7) of the DEGs in DBA/2J under the GO term “ECM organization” were found in the *Yap* cKO dataset as well [38] (Supplementary Fig. 3B). Taken together, this transcriptomic analysis uncovers the molecular signature of *Yap* cKO retinas, and highlights ECM organization as a key dysregulated process.

## Discussion

**Fig. 8:**
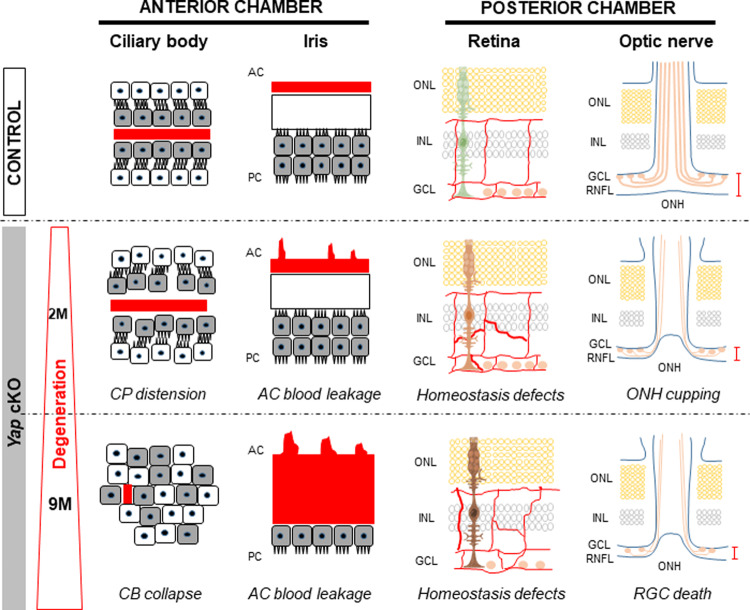
Uveitic Glaucoma-like features in *Yap* cKO mice. *Yap* deletion in both Müller glia and NPE cells of the ciliary body leads to defects in homeostatic key functions. This eventually results in the development of a glaucoma-like phenotype. Within the anterior chamber, *Yap* cKO mice harbor breakdown of the blood-aqueous barrier starting by 2 months (2 M) of age at the iris level, and distension of ciliary processes. The ciliary body then further degenerates until complete collapse at 9 months (9 M) of age. At the retinal level, early defects include downregulation of Müller cell proteins like GS, vascular defects, and optic nerve head deformation. This is followed along with aging by reactive gliosis, deregulation of glutamate homeostasis and RGC death. AC anterior chamber, CB ciliary body, CP ciliary processes, GCL ganglion cell layer, INL inner nuclear layer, ONH optic nerve head, ONL outer nuclear layer, PC posterior chamber, RGC retinal ganglion cells, RNFL retinal nerve fiber layer.

The mechanisms underlying the pathogenesis of neurodegeneration in glaucoma are still poorly understood. We here document a uveitic glaucoma-like phenotype resulting from *Yap* deletion in both the ciliary body NPE and Müller cells (Fig. 8). In the anterior chamber of *Yap* cKO eyes, we identified early BaB breakdown and ciliary body collapse, phenocopying patients with uveitis. In the neuroretina, we highlighted early vascular defects and optic nerve head cupping, as well as Müller cell dysfunctions that worsen with age (including sustained reactive gliosis and glutamate recycling defects). Ultimately, old *Yap* cKO mice start exhibiting RGC loss. We also provide a list of early-deregulated genes (including direct YAP target genes) that could contribute to the observed phenotype. Altogether our results reveal that *Yap* cKO mice harbor many of the important features observed in glaucomatous patients with uveitis. YAP is now recognized as a crucial mediator of eye development, homeostasis and disease [10, 44, 45]. Besides its major functions as a cell-autonomous regulator of key cellular processes (proliferation, differentiation, apoptosis…), YAP also acts at the level of the tissue structure by bridging extracellular matrix changes to the cell through its mechanosensory activity [46], and by contributing to the establishment and maintenance of cellular junctions within epithelia [47]. It is thus important to stress out that the conditional deletion of *Yap* probably results in profound changes within surrounding tissues, through deregulation of local microenvironments. In line with this, our RNAseq analysis suggests a major disorganization of the ECM within *Yap* cKO retinas, which precedes the onset of the retinal glaucomatous phenotype. Noteworthy, we identified FN1 as being the most significant hub, a trait reminiscent of the observed situation in the trabecular meshwork of glaucomatous patients [40]. Since *Fn1* is a direct transcriptional YAP target, it would be worth investigating whether the increase of FN1 in glaucomatous patients is due to a deregulation of YAP.

In the anterior chamber, the deletion of *Yap* in NPE cells results in ciliary body defects that worsen with age. The early ciliary process distension we observed is indicative of cell-cell contact rigidity loss and is consistent with the known requirement of YAP in cellular junction integrity [47–49]. This is followed by progressive embedment of NPE cells inside the collapsed ciliary body. It was previously shown that gap junction integrity between NPE cells and the pigmented epithelial cells is necessary for the control of IOP [50]. Of note, although both *Yap* cKO males and females harbored collapsed CB, only the latter exhibited enhanced IOP values. Noticeably, such gender-specificity IOP defects have already been described in the DBA/2J model, a model resulting from mutations in two genes expressed in the iris and the ciliary body, *Gpnmb* and *Tyrp1* [23, 24]. In this model, the IOP increases earlier (around 6 months compared to 8 months) and remains higher for a longer period of time in females than in males [23, 25]. Future studies should explore the underlying factors contributing to this gender disparity in both DBA/2J and *Yap* cKO mice. Apart from the alteration of the optic nerve head, the phenotypic evolution of *Yap* cKO eyes resembles the one observed in DBA/2J mice on several other aspects. In both cases, disruption of the iridal BaB precedes the complete collapse of the ciliary body [23, 24]. Since in our model, *Yap* is not deleted in the iris, the observed blood leakage is likely an indirect consequence of NPE dysfunction. Based on the intimate topological connection between the iris and the ciliary body, an attractive hypothesis would be that mechanical perturbations generated by the disruption of NPE integrity might be transmitted through the iris stroma to the endothelial cell layer, eventually leading to iridal vascular damage.

At the neuroretinal level, *Yap* cKO mice harbor several defects that likely contribute to their glaucoma-like phenotype. In particular, we observed loss of retinal blood vessel integrity, as well as Müller cell dysfunctions related to water clearance, ion transport, and glutamate recycling. Deregulation of these essential intertwined pathways starts early in *Yap* cKO retinas as inferred by our whole transcriptome analysis, and it is expected to compromise retinal homeostasis. The water transport is linked to ion fluxes and thereby contributes to neuro-glial communication and neuronal electrical activity. In particular, the coupling of AQP4 with potassium channels like Kir4.1 ensures the establishment of proper K^+^ gradients that are crucial for RGCs physiology [51, 52]. The decreased expression of both proteins observed in aged *Yap* cKO Müller glia may thus participate in the onset of RGCs degeneration. Glutamate homeostasis disruption probably represents another important contributor to RGC death. Indeed, under healthy conditions, Müller glia ensures glutamate recycling through its uptake and subsequent transformation into either glutamine, to fuel neurons, or glutathione antioxidant [32]. The altered glutamate to glutamine ratio exhibited by *Yap* cKO retina probably originates from the downregulation of glutamine synthetase expression, and from the altered expression of key glutamatergic genes, that occurs as early as two months of age. We did not observe elevated levels of glutamate in retinal extracts, but we do not exclude that free glutamate might be increased due to impaired reuptake. This function is ensured by the Glutamate-Aspartate Transporter (GLAST) expressed in Müller glial cells. Since its activity is highly dependent on potassium buffering by the Kir4.1 channel [53], it might thus be affected by the severe decreased expression of this protein in aged *Yap* cKO retinas. Under this hypothesis, glutamate reuptake may not be optimal and could therefore contribute to local excitotoxicity and eventually RGC death. Of note, such a decreased Müller cell expression of Kir4.1 has been described in glaucomatous patients [54]. Finally, a growing wealth of clinical data supports the role of vascular dysfunction in certain cases of glaucoma [31]. However, as most clinical studies have been performed in well-advanced glaucomatous patients, it remains unclear whether these vascular defects occur before RGC death, or whether they are a consequence of their degeneration. In *Yap* cKO mice, we observed that RGC loss is not only preceded by reactive gliosis but also by the appearance of severe retinal vascular defects. These might affect RGC survival by inducing ischemia and subsequent oxidative stress. *Yap* cKO mice could therefore represent a useful tool for identifying the chain of events leading to such neuro-glia vascular unit dysfunctions and help to better apprehend how the interplay between different stressors leads to RGC degeneration.

Glaucoma is multifactorial and therefore involves multiple and intermingled mechanisms. No uveitis and/or glaucoma model will thus recapitulate the full spectrum of clinical features described in patients [55]. Nonetheless, *Yap* cKO mice offer the dual advantage of providing both a model of uveitis in young individuals, and a model for deciphering the mechanisms that progressively lead to RGC death along with age. In particular, the similarities we highlight between *Yap* cKO mice and uveitic patients in terms of ciliary body defects support the use of *Yap* cKO mice as a relevant model to study the mechanisms underlying ciliary body degeneration. This is of utmost importance considering the high prevalence (around 20%) of glaucoma complications among patients with uveitis [56] and the lack of early diagnosis biomarkers [57]. We believe that *Yap* cKO mice could help identifying such biomarkers, thereby allowing a better management of glaucomatous patients.

Several genes associated with diverse types of glaucoma were identified in our retinal RNAseq analysis, reinforcing the fact that the *Yap* cKO phenotype bears strong similarities with the human pathology. Our mouse line thus offers a complementary model to the gold standard congenital glaucoma DBA/2J mice. Importantly, the targeted deletion of *Yap* in the CB and Müller cells avoids lethal systemic complications, such as cardiac calcification or aortic aneurism, which are observed in 22% of DBA/2J mice [58]. Nevertheless, some limitations remain. First, the deletion of *Yap* in two different cell types, precludes the unambiguous attribution of a phenotypic trait to the dysfunction of one or the other tissue. Development of Müller glia- or CB-specific cKO models is now required for a better understanding of the cause-and-effect relationships. Second, although the vast majority of *Yap* cKO glaucoma features are consistent from one mouse to another, IOP measurements were highly variable in the colony. This variability is inherent to the tonometer usage and has also been observed in the DBA/2J colony, with fluctuations in terms of onset and amplitude [59–61]. Therefore, the IOP analysis should be reinforced in future experiments using intracameral measurements. Finally, it would be insightful to monitor RGC function by evaluating the optic nerve head component of visual function through second-order kernel response determination by electroretinography [62].

In conclusion and despite the aforementioned limitations, our data bring the first experimental evidence pointing to YAP as a key player in glaucoma pathogenesis. They also highlight *Yap* cKO mice as a preclinical model that might prove useful to elucidate how anterior uveitis may favor glaucoma development, and to investigate in the glaucomatous phase, how glial and vascular perturbations ultimately result in RGC death. More importantly, this model can offer a new opportunity to test therapeutic approaches targeting this disease.

## Materials and methods

### Animals

*Yap*^*flox/flox*^ and *Yap*^*flox/flox*^;*Rax-CreER*^*T2*^ mice [16] were kept at 21 °C, under a 12-hour light/12-hour dark cycle, with food and water supplied *ad libitum*. Cre recombinase activity was induced through a double intraperitoneal injection of 4-hydroxytamoxifen (4-OHT; 1 mg) at P10 and P14. Of note, the entire progeny was injected with 4-OHT with genotype blinding at the time of injection. Genotyping was performed on tail snip genomic DNA as previously described [16]. Genotypes and genders of littermates fit with the expected fifty/fifty ratios. No randomization and no blinding were used. Sample size was chosen to ensure adequate power of statistical tests.

### Retinal dissection

The eyes of sacrificed animals were rapidly enucleated and dissected in Hank’s balanced salt solution (HBSS, Fisher Scientific, Illkirch Graffenstaden, France). A hole was done with a 33-gauge needle just below the ora serrata. Scissors were inserted horizontally, and a cut was performed all the way below the ora serrata to separate the anterior chamber from the posterior one. The lens was gently removed with forceps and the retina was picked up at the basis of the optic nerve and immediately frozen until further experiments.

### RNA sequencing

#### RNA extraction and library preparation

Whole transcriptome analysis was performed on three independent biological replicates from 2-month-old control (*Yap*^*flox/flox*^) and *Yap* cKO (*Yap*^*flox/flox*^*;RaxCre*^*ERT2*^) retinas. Harvested retinas were snap frozen before RNA extraction using the RNeasy Mini Kit (Qiagen). RNA quality and quantity were evaluated using a BioAnalyzer 2100 with RNA 6000 Nano Kit (Agilent Technologies). Stranded RNA-Seq libraries were constructed from 100 ng of high-quality total RNA (RIN > 8) using the TruSeq Stranded mRNA Library Preparation Kit (Illumina). Paired-end sequencing of 40 bases length was performed on a NextSeq 500 system (Illumina).

#### Bioinformatical analysis

Pass-filtered reads were mapped using STAR and aligned to mouse reference genome GRCm38.94 [63]. Count table of the gene features was obtained using FeatureCounts [64]. Normalization, differential expression analysis and TPM (Transcripts Per Million) values were computed using EdgeR [65]. An FDR of less than or equal to 0.05 was considered significant. A cutoff of 1.5 for fold change and a minimum expression of 5 TPM in one experimental group were applied to filter out differentially expressed genes. Comprehensive gene list analysis, enriched biological pathways and gene annotation, were based on the Gene Ontology classification using Metascape [66]. Data visualization was done using GOplot R package [67]. Additionally, the list of deregulated genes (DEGs) in our *Yap* cKO dataset was compared to the list of DEGs in a RNAseq DBA/2 J dataset (comparison of 3-month-old C57BL/6 J and DBA/2 J retinas, GSE127942 [68]), and to the list of YAP direct target genes retrieved from the Harmonizome database (inferred from integrating genome-wide ChIP-X experiments) [37, 69]. The protein-protein interaction network was constructed using the String database of known and predicted protein-protein interactions, with the highest confidence score ≥ 0.9 [70]. The open-source software Cytoscape version 3.10.1 (https://www.cytoscape.org) was used to determine the node degree for each DEGs.

### Histology and immunofluorescence

Immunostaining on sections was performed using standard procedures [16]. Briefly, enucleated eyes were fixed in chilled 1X PBS, 4% paraformaldehyde (PFA) for 20 min at 4 °C. A hole was done with a 33-gauge needle at the ora serrata and the eyes were fixed for another 30 min in PFA. Fixed samples were dehydrated, embedded in paraffin, and sectioned with a Microm HM 340E microtome (Fisher Scientific). Antigen unmasking treatment was done in boiling heat-mediated antigen retrieval buffer (10 mM sodium citrate, pH 6.0) for 20 min. Section permeabilization was performed in PBS 0.3% Triton X-100 for 7 min at RT. All primary and secondary antibodies are listed in Supplementary Table S3. Nuclei were counterstained with DAPI (1 mg/ml, Fisher Scientific) for 10 min.

For NEFL immunostaining on flat-mounted retinas, retinas were dissected in HBSS and all the vitreous was removed to optimize antibody binding. The retinas were then fixed in 4% PFA for 20 min, washed with PBS, and permeabilized/blocked in DAKO diluent (Agilent, Les Ulis, France) containing 0.5% Triton X-100 for 7 min at RT. The same solution was used for incubation in primary and secondary antibodies. Four radial incisions were then made in the retina to create a petal shape and allow flat mounting with ganglion cells facing up.

### Imaging

Fluorescence and brightfield images of paraffin-embedded eye sections were acquired using an ApoTome-equipped AxioImager.M2 microscope. Image processing was performed using Zen (Zeiss, Rueil Malmaison, France), and ImageJ softwares [71]. The same magnification, laser intensity, gain, and offset settings were used across animals for any given marker. Ultrasound biomicroscopy images were acquired using a Reflex UBM (Reichert Technologies, München, Germany).

### Western blot

Western blot was performed on proteins extracted from a single retina [16]. Between seven and sixteen individuals were tested per condition. Harvested retinas were snap frozen before lysis in 80 μL P300 buffer (20 mM Na_2_HPO_4_; 250 mM NaCl; 30 mM NaPPi; 0.1% Nonidet P-40; 5 mM EDTA; 5 mM DTT) and protease inhibitor cocktail (Sigma-Aldrich, Saint-Quentin Fallavier, France). Protein content was measured using the Lowry protein assay kit (DC Protein Assay; Bio-Rad, Marnes-la-Coquette, France). Homogenates were sonicated and centrifuged for 15 min at 5 000 g, and then 10 μg of the supernatant were subjected to SDS-PAGE, as previously described [12]. Western blots were then conducted using standard procedures. Primary and secondary antibodies are listed in Supplementary Table S3. Antibody binding was revealed by the Enhanced Chemiluminescence System (Bio-Rad) on X-Ray film (Sigma-Aldrich). Each sample was probed once with anti-α-tubulin antibody for normalization. Quantification was done using ImageJ software [71]. All original western blot gels are depicted in Supplementary Fig. 4.

### Measurement of the Glutamate-to-Glutamine ratio by liquid chromatography coupled to high-resolution mass spectrometry (LC-HRMS)

Retinas were resuspended in 170 µL of ultrapure water and then sonicated 2 times for 10 s using a sonication probe (Vibra cell, Fisher Scientific). At this step, 20 µL of each sample were withdrawn for total protein concentration determination (Pierce BCA Protein Assay Kit, Fisher Scientific). Then, we added to the remaining lysate 350 µL of methanol and 10 μL of an internal standard mixture containing 50 µg/mL of ^13^C_5_-glutamine (Eurisotop, Saint-Aubin, France) and ^13^C_5_-glutamic acid (Sigma-Aldrich). Following a 1.5-hour incubation on ice, cell debris were removed by centrifugation for 15 min at 4 °C and 20 000 g. The resulting metabolic extracts were dried under a stream of nitrogen using a TurboVap instrument (Fisher Scientific) and stored at −80 °C until analysis. Dried extracts were dissolved in 30 μL of mobile phase A (see below), sonicated, vortexed, and mixed with 70 µL of mobile phase B, sonicated and vortexed again before a final centrifugation step at 4 °C for 5 min at 20,000 g. The resulting supernatants were then transferred into vials for analysis by LC-HRMS.

LC-HRMS quantification of glutamine and glutamic acid was performed using a Dionex Ultimate chromatographic system (Fisher Scientific) coupled to a Q-Exactive Plus mass spectrometer (Fisher Scientific) equipped with an electrospray ion source. The mass spectrometer was externally calibrated before each analysis according to the manufacturer’s instructions. Chromatographic separation was performed on a Sequant ZIC-pHILIC column (5 μm, 2.1 × 150 mm; Merck, Fontenay-sous-Bois, France) maintained at 45 °C. Mobile phase A consisted of an aqueous buffer of 10 mM of ammonium acetate, while mobile phase B was made of 100% acetonitrile. Chromatographic elution was achieved at a flow rate of 200 μL/min. After injection of 10 μL of the sample, elution started with an isocratic step of 2 min at 30% A, followed by a linear gradient from 30 to 60% of phase A during 5 min. The chromatographic system was then rinsed for 5 min at 0% B, and the run ended with an equilibration step of 8 min. The column effluent was directly introduced into the electrospray source of the mass spectrometer, and analyses were performed in the negative ion mode. Source parameters were as follows: capillary voltage, -2.5 kV; capillary temperature, 350 °C; sheath gas and auxiliary gas pressures set at 35 and 10 arbitrary units, respectively. The detection was performed from m/z 50 to 600 at a resolution of 70 000 (full width at half-maximum at m/z 200) using an AGC target of 1e6 and a maximum injection time of 250 ms. Glutamine and glutamic acid were detected as deprotonated [M-H]- species at m/z 145.0618 and m/z 146.0458, and elute at 4.7 and 4.8 min, respectively. Both entities were quantified by isotope dilution using their corresponding ^13^C-labeled homologs as internal standards and concentrations expressed as µg of glutamine or glutamic acid per gram of total proteins.

### Measurement of intraocular pressure (IOP)

IOP was measured in both eyes with a rebound tonometer (Tono-Lab, Icare Tonovet, Vantaa, Finland) [72] on 6- and 9-month-old awakened mice. At each time point, 18 consecutive readings were made for each eye and averaged. To limit fluctuations of the IOP due to the circadian rhythm, we always measured IOP in the morning [72].

### Retinal and anterior chamber angiography

Mice were anesthetized with an intraperitoneal injection of ketamine (90 mg/ kg, Merial) and xylazine (8 mg/kg, Bayer, MüllerStrasse, Berlin, Germany). Fluorescein angiography was performed by injecting 150 μL dextran-conjugated fluorescein (25 mg/ml final, molecular weight [MW] 10 000) into the vein tail of anesthetized mice. Images were taken for up to 5 min post-injection after pupil dilation by topical application of tropicamide (0.5%) and phenylephrine (2.5%) for retinal images, or without pupil dilation for anterior chamber images, using a Micron IV retinal imaging microscope (Phoenix Research Labs, Pleasanton, California, USA).

### Optic coherence tomography (OCT)

Mice were anesthetized with an intraperitoneal injection of ketamine (90 mg/ kg, Merial, Lyon, France) and xylazine (8 mg/kg, Bayer). Pupils were dilated by topical application of tropicamide (0.5%) and phenylephrine (2.5%). OCT was performed using the R2200 Spectral Domain OCT system Imaging System (Bioptigen, Leica Microsystems, Nanterre, France). For optic nerve head imaging, a general mouse OCT lens was used with a reference arm set to 1 020. A rectangular volume analysis was performed, using 33 consecutive B-scans lines, each one composed of 1000 A-scans and three frames. The volume diameter was 1.4 mm.

### Ultrasound biomicroscopy (UBM)

To visualize the anterior chamber angle including the ciliary body and ciliary processes, a UBM examination was performed in patients in the supine position, by one operator, in mesopic conditions, after topical anesthesia with oxybuprocaine hydrochloride (Anestocil®) with a Reflex UBM (Reichert Technologies). The plastic shell was preferred to prevent globe distortion, and immersion was accomplished with distilled water and Hypromellose with Carbomer 980 (Genteal Tears, Alcon, Vernier-Geneva, Switzerland) applied along the shell´s interior rim to keep the bath sealed. Patients were instructed to gaze straight ahead at one point in the ceiling to acquire as close as possible the axial sections. For transverse sections, patients were asked to gaze in the opposite direction of the meridian under observation, and the ultrasound probe was placed over the latter with its marker towards up or nasally for a transverse swipe. Pathology photographs were obtained with the verbal consent of the patients and de-identified afterwards.

### Statistics

All data were analyzed using GraphPad Prism version 8.3 for Windows (GraphPad Software, La Jolla California USA; www.graphpad.com). Since Gaussian distribution did not prove true (Shapiro-Wilk test) for all datasets, comparisons involving two unpaired groups were analyzed using the non-parametric two-tailed Mann-Whitney test. A *p* value < 0.05 was set as the basis for rejecting the null hypothesis. Sample sizing is depicted in the figure legends.

### Supplementary information

Supplemental Figure 1

Supplemental Figure 2

Supplemental Figure 3

Supplemental Figure 4

Supplemental Table S1

Supplemental Table S2

Supplemental Table S3

## Data Availability

The raw RNA sequencing data are freely accessible in the Gene Expression Omnibus (GEO) database under the accession number GSE251685. The full list of DEGs (FDR ≤ 0.05, FC ≥ 1.5, TPM ≥ 5), and the Gene Ontology analysis are available in the supplemental material of the manuscript.
