# Supplementary figures and images for: Uveitic glaucoma-like features in Yap conditional knockout mice
Source: Cell Death Discov. 2024 Jan 25;10:48. doi: 10.1038/s41420-023-01791-6 (PMC10811226; doi:10.1038/s41420-023-01791-6)

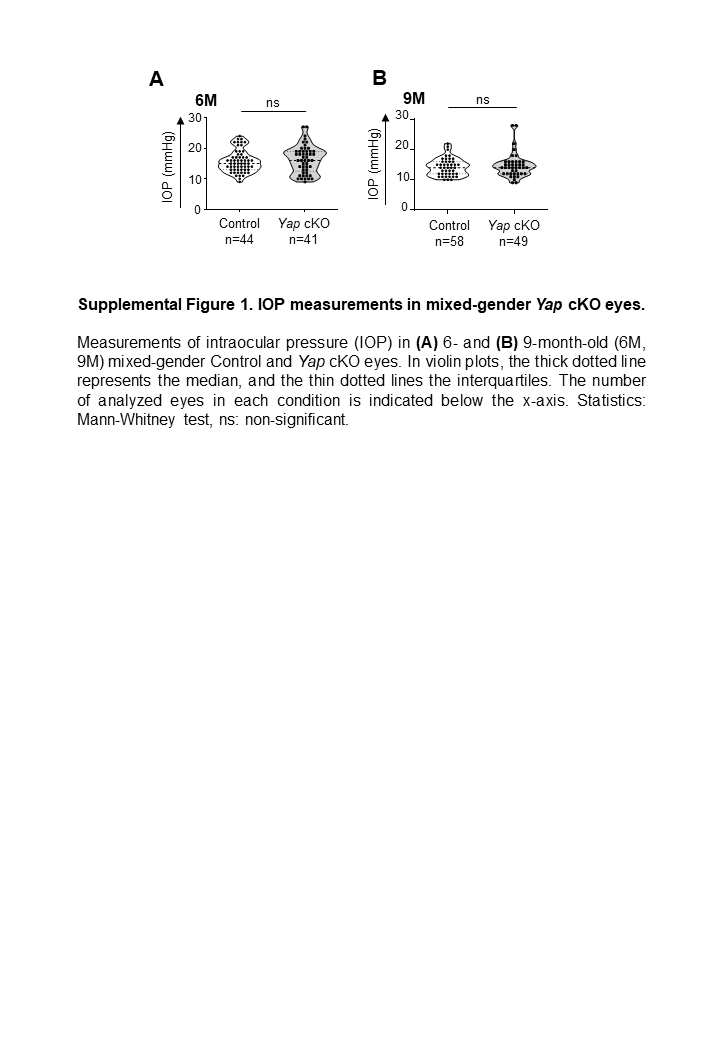

Supplement: Supplementary file 1 — Supplemental Figure 1 [file 41420_2023_1791_MOESM1_ESM.tif]

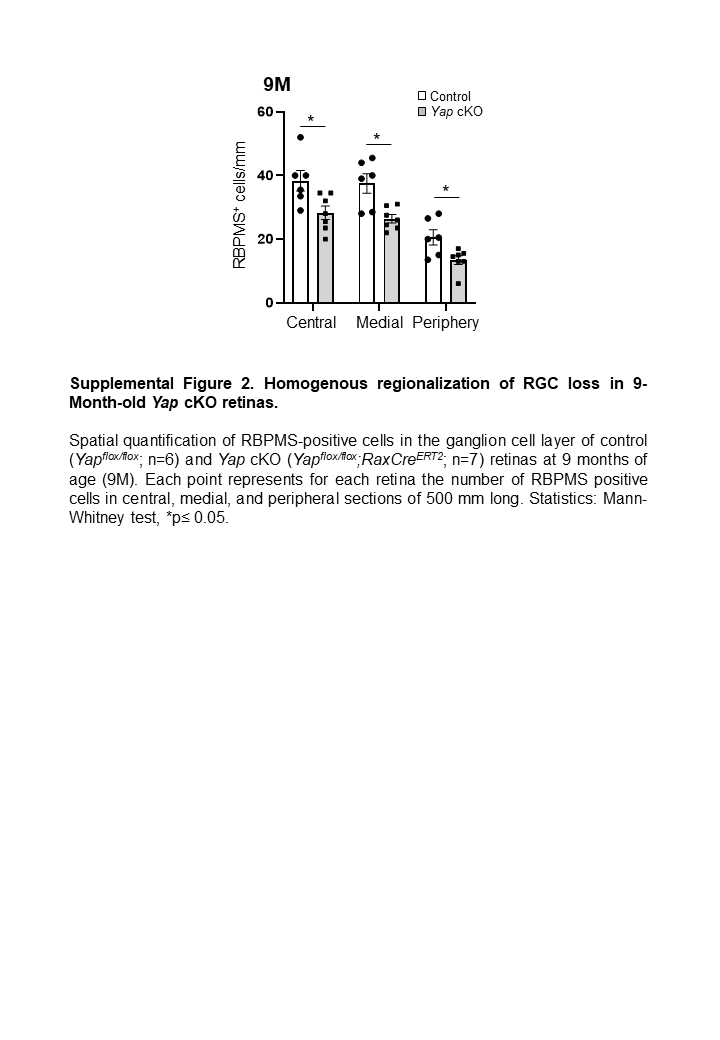

Supplement: Supplementary file 2 — Supplemental Figure 2 [file 41420_2023_1791_MOESM2_ESM.tif]

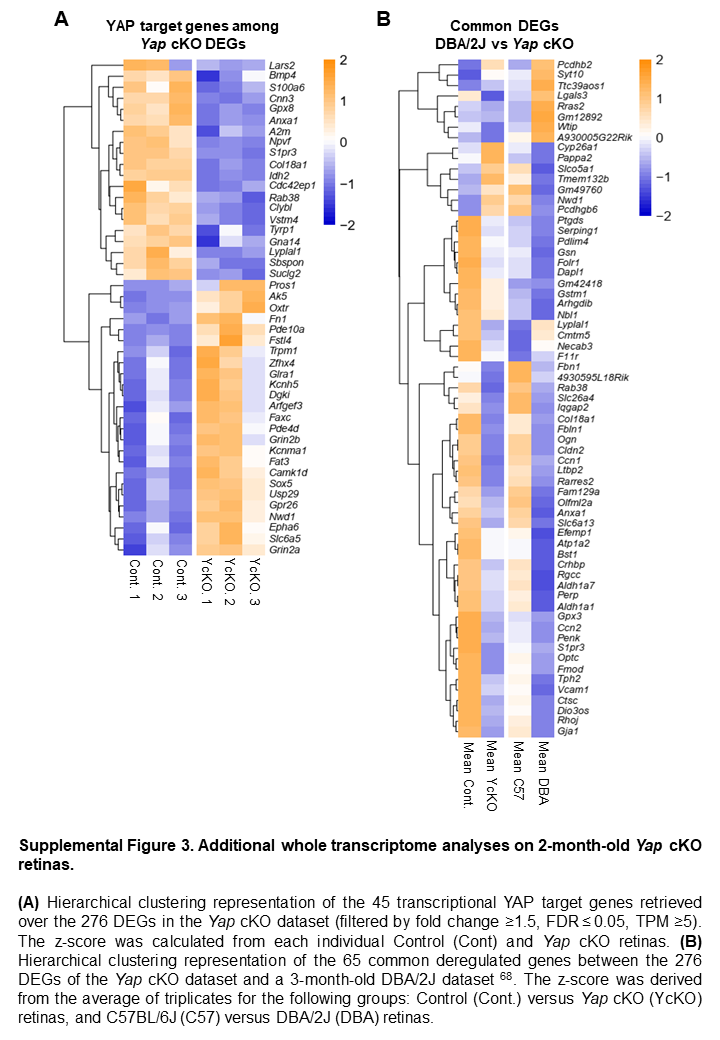

Supplement: Supplementary file 3 — Supplemental Figure 3 [file 41420_2023_1791_MOESM3_ESM.tif]

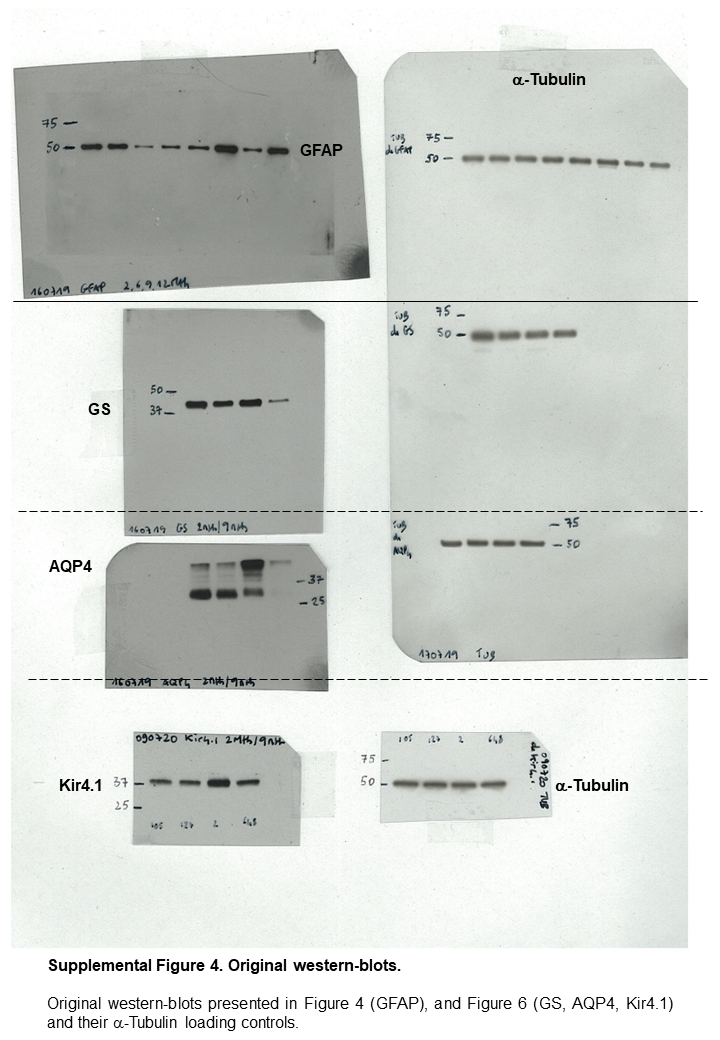

Supplement: Supplementary file 4 — Supplemental Figure 4 [file 41420_2023_1791_MOESM4_ESM.tif]
